# Animal Models of Epilepsy: A Phenotype-oriented Review

**DOI:** 10.14336/AD.2021.0723

**Published:** 2022-02-01

**Authors:** Yilin Wang, Penghu Wei, Feng Yan, Yumin Luo, Guoguang Zhao

**Affiliations:** ^1^Department of Neurosurgery, Xuanwu Hospital of Capital Medical University, Beijing, China.; ^2^Institute of Cerebrovascular Diseases Research and Department of Neurology, Xuanwu Hospital, Capital Medical University, Beijing, China.; ^3^Beijing Institute for Brain Disorders, Capital Medical University, Beijing, China.; ^4^Clinical Research Center for Epilepsy Capital Medical University, Beijing, China

**Keywords:** epilepsy, animal models, seizure phenotype, kindling model, temporal lobe epilepsy

## Abstract

Epilepsy is a serious neurological disorder characterized by abnormal, recurrent, and synchronous discharges in the brain. Long-term recurrent seizure attacks can cause serious damage to brain function, which is usually observed in patients with temporal lobe epilepsy. Controlling seizure attacks is vital for the treatment and prognosis of epilepsy. Animal models, such as the kindling model, which was the most widely used model in the past, allow the understanding of the potential epileptogenic mechanisms and selection of antiepileptic drugs. In recent years, various animal models of epilepsy have been established to mimic different seizure types, without clear merits and demerits. Accordingly, this review provides a summary of the views mentioned above, aiming to provide a reference for animal model selection.

Worldwide, 65 million people experience epilepsy, which is an abnormal nervous transient dysfunction that affects motor, sensory, consciousness, and autonomic nervous system [1], usually accompanied by abnormal electroencephalogram (EEG) and pathological abnormalities [2]. Recurrent seizure attacks can cause neuronal damage and necrosis [3], cerebral dysfunction aggravation, and life-threatening effects, posing a heavy burden on the patients’ families [4]. Animal models of epilepsy are conducive to the selection of antiepileptic drugs (AEDs) and the development of new therapies, significantly promoting the process of epilepsy-related studies [5]. Almost all animals with a central nervous system are likely to experience epilepsy, which contributes to a wide range of animal model species. Among animals, dogs and cats have been prioritized over the past few years. Recently, rodents have increasingly been considered a popular species for epilepsy animal models, especially those involving gene mutations and genetically modified technology, although they can also be established in other animals, such as primates. Except for rats, dogs are also appropriate for epilepsy model establishment [6]. Approximately 30% of the patients with epilepsy are suffering from drug-resistant epilepsy [7]. Drug-resistant epilepsy models have been successfully established in dogs by Jambroszyk et al. and have been proven to be suitable for assessing the therapeutic effect of AEDs [8]. Additionally, zebrafish for epilepsy models has been increasingly popular because of their simpler breeding and maintenance requirements, as well as higher fertility, compared with rodents [9].

The predominant species and inducing methods for animal models of epilepsy are rodents and kindling, respectively. Classical kindling is the repeated electrical stimulation of limbic structures that leads to abnormal discharge and seizure attacks [10]. Kindling is mainly divided into electrical and chemical types. Electrical kindling refers to the deep electrode implanted into the limbic brain region, e.g., the amygdala and hippocampus, or the topical stimulation of the corneas [11]. Conversely, chemical kindling refers to the systematic injection of excitatory matter into the brain, leading to seizure attacks [12].

Currently, no model can cover all the seizure phenotypes and perfectly mimic the seizure characteristics of humans. Accordingly, given the complexity of seizure characteristics, the selection of models may be based on concrete needs in practice. Previous studies have summarized different animal models based on different induction methods, aiming to describe the details of manufacturing operations. To avoid repetition, we reviewed the existing animal models according to different seizure phenotypes with a focus on the merits and demerits, aiming to provide a reference for animal model selection.

## Generalized tonic-clonic seizure models

Maximal electroshock (MES) models are referred to as electrical stimulations through corneal or auricular electrodes, which are frequently used to mimic tonic-clonic seizure attacks [13, 14]. Compared with transcorneal stimulation, transauricular stimulation is more effective in inducing tonic convulsions with a lower threshold. However, the face and forelimb clonus cannot be induced using transauricular electrodes but can be achieved using transcorneal electrodes [13]. MES models are appropriate for the preclinical identification of anticonvulsive properties of the tested compound. The specific dose and application time of AEDs should be guided by previously published literature and experimental experiences [15]. The tested AEDs are commonly administered 1 h before electrical stimulation [14]. Currently, many new AEDs and newly developed therapies have been identified using MES models [16, 17].

Pentylenetetrazol (PTZ) couples to the γ-aminobutyric acid (GABA)A receptor, serving as a potential chemical epileptogenic agent [18]. Caudal vein injection [19] or intraperitoneal injection of PTZ [20] can induce tonic-clonic seizures in rats. The rats in the PTZ models present involuntary movements of the head, forelimbs, or hind limbs and subsequently progress to falling, clonic convulsion, and complete limb extension [20]. Both the MES and PTZ models were easily established using a simple procedure. In addition, the MES model is considered pharmacologically independent, whereas the PTZ model may be involved in the interaction with the tested AEDs [13, 14, 16, 19-21]. However, the literature has indicated that both MES and PTZ models belong to acute seizure models, and thus, cannot serve as resistant seizure models [21]. More importantly, the tested compounds may have different therapeutic activities in different models. Thus, the model discrepancy should be considered when evaluating the tested drugs [22].

Flurothyl is a GABAA antagonist with epileptogenic ability [23]. Acute generalized tonic-clonic seizure mouse model can be established by flurothyl inhalation [24-26], which manifests as convulsions and tonic extension of the limbs [27, 28]. The rats with recurrent seizures induced by flurothyl usually present reflex delay, poor adaptation to new environments, and poor memory and learning abilities [27]. A previous study revealed that the classical pathological changes in flurothyl-kindling mice with Angelman syndrome were perineuronal net deposition in the dentate gyrus without obvious mossy fiber sprouting or neuronal loss [29]. Given its high volatility, flurothyl can trigger seizure attacks by inhalation without injection and clear up quickly without any potential remaining effects in the body. Moreover, seizures can be easily controlled by stopping the inhalation of flurothyl and switching to air inhalation. Accordingly, the seizure duration is relatively short [23]. In addition, the latency and sensitivity to seizure occurrence vary by genotype and age in flurothyl mouse models [30, 31].

N-methyl-D-aspartic acid (NMDA) serves as a common excitatory epileptogenic agent that induces a cellular excitotoxicity state with a massive calcium influx and hippocampal network hyperexcitability [1]. In the past, exogenous administration of NMDA receptor agonists in the rodent brain successfully induced seizure attacks [32]. Studies have established generalized tonic-clonic seizure models by injection through the vitreous cavity or intraperitoneal injection of NMDA in zebrafish [1]. The main behavioral changes consist of leaping, quick movement, swimming in circles, and a series of overresponses to stimuli [1].The model is also equipped with a simple procedure, but with a single epileptogenic mechanism and extracranial organ injury, such as the heart [1].

Fluid percussion injury (FPI) models are implemented by exposing the intact dura mater to a fluid pressure pulse through craniotomy and a closed hydraulic system [33], leading to neuronal injury and seizure attack [34]. FPI models are also widely used in the study of posttraumatic epilepsy (PTE) [35], dividing them into midline, parasagittal, and lateral models based on the position of the craniotomy away from the sagittal suture [36]. Studies have reported that FPI can induce generalized tonic-clonic seizures [37]. FPI mimics the characteristics of human traumatic brain injury (TBI), such as intracranial hemorrhage, cerebral edema, and gray matter damage [36]. After FPI in the acute phase, pinnal, corneal, paw flexion, and righting responses disappear temporarily and reoccur quickly [35]. After the acute phase, cognitive impairment, movement, and memory disorders can be observed for at least a year after severe FPI [35, 36]. Classical pathological changes include brain edema, intracranial hemorrhage, focal contusion in the cortex, progressive gray matter injury, diffused white matter damage, and tears [35]. In the long term, progressive degeneration may occur in the ipsilateral hippocampus, thalamus, amygdala, and striatum [36]. The main advantages of the FPI models are the need for simple equipment and pathophysiological replication of human TBI, such as intracranial hemorrhage, brain swelling, and persistent neuromotor and cognitive deficits. However, precise surgical progress is vital, given that a slight deviation can easily cause high variability. In addition, the model lacks translation to therapeutic predictive validity [33-38]. Nonetheless, other studies have indicated FPI models as potential candidates for drug-resistant epilepsy models, contributing to preclinical drug development [39]. Besides, lateral mild FPI model can also be used for new therapy development in neurodegenerative disorders [40].

The impact-acceleration model, which is also known as the weight drop model [33], refers to the specific weight dropping from a specific height above the naked dura mater [35]. This model can cause severe brain injury and induce generalized tonic-clonic seizures [35], which is widely used in the study of brain hyperexcitability after trauma [34], and may also cause early convulsions with a mild-to-moderate degree in the acute stage, and even late convulsions persisting up to 15 weeks after injury [33]. Morphologically, immediate white matter hemorrhage below the contused cortex in the first hour post-injury can be observed, which gradually evolves into a necrotic cavity after 24 h and expands over the next 2 weeks. Regarding neurofunctional deficits, most can recover in the first 2 weeks post-injury, whereas for severe contusions, deficits can persist for at least 3 months [36]. This protocol is simple and inexpensive, and the degree of brain damage varies by the dropped weight and height; thus, the injury severity is controllable [36]. However, high mortality rates, low reproducibility, and transient spontaneous seizures may be considered.

Closed-head injury (CHI) models are established using projectiles impacting the surface of the skull [18]. Studies have established CHI models in rats [18] and zebrafish [41], with a successful induction of generalized tonic-clonic seizure in the zebrafish CHI model [41]. Zebrafish are equipped with strong fertility and a cost-efficient raising condition. Compared with rodents, zebrafish models present rapid seizure attacks, short latent periods, and high rates of spontaneous recurrent seizures [41]. The CHI model also presents a reliable onset without craniotomy [18, 41]. In a previous mouse CHI model, neurological impairment and behavioral deficits have been reported, and neurodegeneration, astrocyte and microglia activation, blood-brain barrier breakdown, and abnormal morphology on MRI have also been observed [36]. In recent years, a new CHI model called the Maryland model has been established. It is known to impose an impact force to the anterior part of the skull, generating anterior-posterior and sagittal rotational acceleration of the brain, while leaving the skull intact. Its characteristics include diffuse axonal injury and petechial hemorrhage without skull fracture, cortex contusion, and extended apnea [36].

The iron ion model refers to the intracortical injection of iron ions into the sensorimotor cortex through stereotaxic surgery [42, 43]. Ferrous chloride can promote lipid peroxidation, free radical formation, and abnormal glutamate transportation, mimicking TBI and recurrent PTE attacks [33]. Similarly, ferric chloride (FeCl_3_) can also induce recurrent seizure attacks and epileptic discharges [44]. Intracortical injection of FeCl_3_ into rats has induced generalized tonic-clonic seizure, with the following series of behaviors: face and ear convulsions, axial convulsions through the body, myoclonus and rearing, running and jumping, and generalized tonic-clonic seizures [45]. Spike wave discharges can also be observed in the EEG recordings [44]. Furthermore, FeCl_3_ can increase antioxidant enzyme activity (e.g., superoxide dismutase and catalase), promoting lipid peroxidation in the cortex [44]. The iron ion model presents recurrent seizure attacks with high susceptibility to seizures and neurodegeneration restricted to the cortex. However, stereotaxic surgery is technologically demanding [33, 42-45].

Audiogenic seizures are generalized seizures induced by high-intensity acoustic stimuli [46]. The audiogenic seizure model is also an important genetic epilepsy animal model, which mainly includes Krushinsky-Molodkina rats [47], Genetically Epilepsy-Prone rats, DBA/2 rats [48], and Wistar audiogenic rats [49, 50]. Audiogenically vulnerable animals usually start with wild running and gradually evolve into a tonic-clonic phase [46]. Wistar audiogenic rats model is successfully established [51]. In this model, after exposure to repetitive sound stimuli with high intensity, Wistar audiogenic rats presented wild running, head ventral bending, back arching tonus, and limb hyperextension. The EEG recordings also showed polyspike-wave activity in the amygdala and synchronized epileptiform polyspike activity with high amplitude in the inferior colliculus and cortex [52]. Moreover, audiogenic kindling can induce limbic epileptogenicity and increase hippocampal mitotic rate. No obvious mossy fiber sprouting, or neuronal degeneration was observed [53]. The audiogenic seizure model also presents a high seizure predisposition without the need for craniotomy. The specific trigger (sound stimulation) is necessary to induce seizure attacks without spontaneous recurrent seizures [46].

**Table 1:** Generalized tonic-clonic seizure models.

Model	Animal	Advantages	Disadvantages	References
Maximal electroshock model	Mice	Simple procedure, Pharmacologically-independent, Test AEDs	Cannot serve as a resistant seizure model	[13, 14, 16, 21]
Intraperitoneal injection or caudal vein injection of PTZ	Rodents	Simple procedure, Low cost	Interaction with the tested AEDs	[14, 19-21]
Intravitreal injection of NMDA	Zebrafish	Rapid onset	Difficult microinjection, Complicated ocular pharmacokinetics, Single epileptogenic mechanism	[1]
Inhalation of flurothyl	Rat	No need for injection, No remaining effects, Easily controlled seizure	Short seizure duration, Seizure latency variability, Seizure sensitivity variability	[23, 30, 31]
Intraperitoneal injection of NMDA	Zebrafish	Simple procedure	Extracranial organs injury, Single epileptogenic mechanism	[1]
Fluid percussion injury models	Mouse Cat, rabbit, rat, dog sheep and swine	Simple device, Typical histopathology, High seizure susceptibility, Neuromotor and cognitive deficits	Technologically-demanding, High variability, Craniotomy, High mortality, Long potent period, No therapeutic predictive validity	[33-38]
Impact-acceleration model	Rodent	Simple protocol, Low cost, Controllable injury severity	Low reproducibility, High mortality, Transient spontaneous seizures	[33-36]
The closed head injury models	Mouse Zebrafish	Rapid onset, Reliable onset, Cost-efficiency, No craniotomy	-	[18, 41]
Intracortical injection of iron ion	Mouse	Recurrent seizure attack like PTE, Increased susceptivity to seizure	Stereotaxic surgery	[33, 43-45] [42]
Audiogenic seizure model	Rat	High seizure predisposition, No craniotomy	No spontaneous recurrent seizures	[46]
Optogenetics	Mouse	Reliable onset, No craniotomy, Slight brain injury, cell-type-specific targeted	Repeated stimulation, Animal variability, No spontaneous seizures, Time-consuming	[55, 58] [56, 57]

AEDs, antiepileptic drugs; NMDA, N-methyl-D-aspartic acid; PTE, posttraumatic epilepsy; PTZ, pentylenetetrazol

Generalized epilepsy can also be induced by optogenetics [54, 55], which is characterized by the artificial incorporation of light-sensitive proteins into cell membranes and optical control of neural activity. In the experiments, viral transduction, cell-type-specific expression of light-sensitive channels, and *in vivo* integration of optical stimulation with intracellular and extracellular recording techniques are vital [56]. The potential epileptogenic mechanism of optogenetic mouse models may be related to GABAergic interneurons [57]. The main advantages of the model are its reliable seizure attack, slight brain injury, and cell-type-specific targeting. Conversely, its disadvantages are the absence of spontaneous seizures, species variability, and time consumption due to repeated stimulation [55]. Channelrhodopsins (ChRs) are light-gated, non-specific cation channels that selectively depolarize genetically targeted cells; ChR2 activation through the skull can avoid craniotomy in optogenetic models [58]. Additionally, optogenetics has been shown as a potential candidate for stopping electrographic seizures and reducing the frequency of behavioral seizures in temporal lobe epilepsy [59], focal neocortical epilepsy [60], and intractable epilepsy [61]. The related characteristics of the generalized tonic-clonic models are summarized in Table 1.

## Non-convulsive seizure models

Rats penetrating ballistic-like brain injury (PBBI) models are established by the transmission of shockwaves and projectiles with high energy that penetrate the brain and cause cavity formation, leading to subsequent pathological changes, such as hemorrhage, cerebral edema, gliosis, neuroinflammation, increased intracranial pressure, abnormal blood-brain barrier permeability, cortical spreading depression, and gray and white matter injuries [36]. The corresponding potential neurofunctional outcomes of this model are related to the degree of injury severity, including seizures, sensorimotor impairment, and cognitive deficits [36]. The hallmark of a successfully induced PBBI model is a nonconvulsive seizure attack in early EEG results after brain injury. Given that PBBI models can mimic brain injury caused by gunshot wounds, PBBI models have been widely used in AED selection in early (<7 d) nonconvulsive seizure attacks after brain injury [18].

Explosions are closely related to TBI and PTE. Blast TBI models have been established in rodents and swine [36], which helps in understanding the potential mechanism of PTE [62]. Directly exposing the animals to the shockwave [63] in shock tubes or an open field [33] can establish blast models. Most of the animals present nonconvulsive seizure attacks in successful blast models [63] under the appropriate intensity of shockwaves [38]. Other behavioral abnormalities were also observed, such as impairments in spatial memory, social recognition, and motor coordination [36]. Pathological changes vary according to the severity of blast models, including hippocampal, cerebellar, neocortical, vascular, and axonal injuries and hemorrhage. Few studies have generally assessed related EEG characteristics [33]. The security of explosive storage, strict technical time demand, and environmental conditions limit the widespread use of the blast models, despite the close replication of a real-world blast [33]. The advantages and disadvantages of the PBBI models and blast models are listed in Table 2.

**Table 2:** Non-convulsive seizure models.

Model	Animal	Advantages	Disadvantages	References
PBBI models	Cat Rat	Replication of gunshot wound Injury mechanisms close to TBI	Need standardization	[18]
Blast models	Rodent Pig	Replication of military TBI	Expensive, Risk of explosion, Strict store requirement	[33, 36, 38, 62, 63]

PBBI: penetrating ballistic-like brain injury; TBI: traumatic brain injury

## Absence seizure models

Absence seizures are frequently observed in school-aged children, characterized by a sudden loss of awareness for 3-30 s and interruption of the ongoing behavior, with the classical 2.5-4 Hz spike and slow-wave discharges (SWDs) in EEG [64]. Chemical kindling agents, such as gamma-hydroxybutyrate (GHB) and PTZ, are usually used to establish absence seizure models [65, 66]. GHB, an endogenous molecule, can induce absence seizures by interacting with GABAB receptors. Systemic administration of GHB in Wistar rats can induce behavioral arrest, staring and occasional twitching of the vibrissae, and facial myoclonus, establishing absence seizure models with 4-6 Hz paroxysmal discharges [65]. Intraperitoneal injection of PTZ in rats can induce behavioral features similar to humans, such as behavioral arrest, vibrissae twitching, and facial convulsion with classical bilaterally synchronous SWDs [66]. Both the GHB and PTZ models show typical behavioral changes with SWDs, with high variation [65, 66].

The genetic absence epilepsy rat from Strasbourg (GAERS) model is a popular animal model used to mimic absence epilepsy attack [67], and it is characterized by behavior stagnation and stare [68], with classical SWDs in EEG, which is conducive to the research of potential mechanisms and selection of AEDs [67]. In addition, the GAERS models also present anxiety-like behavior, social impairment, extinction learning impairment, excessive contextual and cued fear conditioning, and dysfunction in crossmodal recognition memory and visual attention [67]. Moreover, because of the similar behavioral manifestations, EEG, and genetic features with humans, the WAG/Rij rat models are widely used in the study of genetic absence seizures, especially in children absence seizures [69]. The WAG/Rij rat usually presents with polypnea, accidental eye-blinks, twitching of the vibrissae, facial myoclonus, and head leaning with classical SWDs [69]. Both the GAERS and the WAG/Rij rat models presented comorbidities and no neurological deficits, which are qualified for testing the targeted AEDs. In addition, more SWDs in the early stage and stable epileptic phenotypes were observed in the GAERS model. The WAG/Rij rat model features reliable seizure onset. Regarding the disadvantages, both were equipped with only one single genotype and no spontaneous remission. Variability in sex and environmental factors may be considered [67-69].

Tottering-6j mice are *Cacna1a* knockout mice that often present with absence-like seizures (e.g., sudden behavioral arrest and staring) and severe ataxia, exhibiting SWDs in the bilateral cortex with relatively higher frequency and shorter duration [70]. The Tottering-6j mouse models are conducive to evaluating AEDs and understanding the pathogenesis of Cav2.1 channelopathies [70]. Table 3 shows the features of the absence seizure models, with merits and demerits.

**Table 3:** Absence seizure models.

Model	Animal	Advantages	Disadvantages	References
Systemic administration of GHB	Cat Rat Mice	SWD Typical behavioral changes	High variation, Species variation	[65]
Intraperitoneal injection of PTZ	Sprague-Dawley rat	Reliably bilateral SWDs, Typical behavioral changes	Sex variability	[66]
GAERS rats	Rat	Test AEDs, More SWDs in early stage, Stable epileptic phenotype, Comorbidities, No neurological deficits	Environmental factors variability, Sex variability, No spontaneous remission, Single genotype	[67-69]
WAG/Rij rats	Rat	Test AEDs, Reliable onset, Comorbidities, No neurological deficits	Environmental factors variability, Age variability, Sex variability No spontaneous remission, Single genotype	[69]
Tottering-6j mouse	Mouse	Test AEDs, Higher SWDs frequency, Cav2.1 channelopathies related mechanism	Shorter SWDs duration	[70]

AEDs, antiepileptic drugs; GAERS, genetic absence epilepsy rat from Strasbourg; GHB, gamma-hydroxybutyrate; PTZ, pentylenetetrazol; SWDs, slow-wave discharges

## Myoclonus models

Myoclonus is characterized by rapid, short, and electric shock-like muscle contraction limited to a single limb or muscle group but occasionally spreads to the whole body [71]. Corneal stimulation for 3 s with a 6-Hz electrical current at 44 mA in mice can induce myoclonus, eventually progressing to generalized convulsions [46, 72]. Common behavioral characteristics include head nodding, forelimb clonus, and jaw clonus [39]. The model is non-invasive, simple, and easy to perform, but with strain variability. In addition, the model can induce a persistent fully kindled state, qualify for a resistant seizure model to evaluate the targeted AEDs [46, 72], and provide guidance for dose selection and development of new therapies targeting drug-resistant epilepsy [39].

Subcutaneous or intraperitoneal injection of PTZ in rodents or birds can mimic human myoclonus [73, 74]. Myoclonus models induced by PTZ are widely used to assess the anticonvulsant effects of AEDs [73, 75]. The disadvantage of this model is dose variability, because an appropriate dose is vital for the successful induction of myoclonus [74]. The related information of the myoclonus models is presented in Table 4.

## Status epilepticus models

Status epilepticus (SE) is a severe emergency, carrying neuronal injury, abnormal neuronal networks and high mortality[76].Common SE models originate from electrical kindling and chemical kindling models [77]. SE can be induced via electrical stimulus in rat models with an electrode implanted into the amygdala or hippocampus [10]. Compared with chemical kindling models, electric kindling models are more suitable for testing AEDs because of the absence of interaction between the proconvulsant and test compounds [77]. Using intrahippocampal electric kindling models, a previous study has shown that adenosine releasing stem cell-derived brain implants 11 can suppress kindling epileptogenesis [78]. Additionally, electric kindling models present a milder phenotype and less severe neuropathologic changes, which are more similar to humans [77]. More importantly, electrical stimulation can rapidly induce SE and immediately stop the electrical stimulus once SE occurs, which cannot be achieved in chemical kindling models [10]. Accordingly, the electrical kindling model presents a relatively lower mortality than chemical kindling models [79]. Stereotaxic surgery is also necessary for the electrical kindling model, and variabilities in age, sex, and strain should be considered [10, 77, 79].

**Table 4:** Myoclonus models.

Model	Animal	Advantages	Disadvantages	References
6-Hz electric corneal stimulation	Mouse	Non-invasive, Resistant seizure model, Persistent fully kindled state, Simple device, Easy procedure, Test AEDs	Strain variability	[46, 72]
Subcutaneous or intraperitoneal injection of PTZ	Rodent Bird	Test AEDs	Dose variability	[73, 75]

AEDs: antiepileptic drugs; PTZ: pentylenetetrazol

Chemical kindling models are classified into subcutaneous, intraperitoneal, or intracerebral injections. Subcutaneous injection or intraperitoneal injection of kainic acid (KA), pilocarpine, or PTZ [80-83], or intracortical injection of KA have successfully established SE models [84]. The common pathological changes of intraamygdala or intrahippocampal injection of KA are neuronal cell loss, astrogliosis, mossy fiber sprouting, granule cell dispersion, and overexpression of the adenosine-metabolizing enzyme adenosine kinase [85]. Regarding the pilocarpine model, prominent thalamic degeneration and enhanced neurogenesis in the granule cell layer of the dentate gyrus have been observed. Additionally, compared with the KA model, more serious neuronal damage is frequently found in the hippocampus, cortex, amygdala, hypothalamus, and endopiriform nucleus [33], and pilocarpine can induce SE rapidly at a low cost, although with higher mortality [86]. The PTZ model also presents dose variability and poor electric activity compared with the pilocarpine model [83].

According to literature, once SE is successfully induced, the occurrence of irreversible injury and neurological deficits in the brain can result in chronic spontaneous recurrent seizures. Accordingly, SE models are candidates for refractory epilepsy models [87]. Given the high mortality of SE models, intraperitoneal injection of diazepam is necessary to interrupt SE and to decrease mortality [88]. Table 5 displays the relevant characteristics of SE models.

**Table 5:** Status epilepticus models.

Model	Animal	Advantages	Disadvantages	References
Electrical stimulation of the hippocampus or amygdala	Rat	Test AEDs, Reliable onset Immediate stop of stimulus, Rapid SE, Lower mortality	Stereotaxic surgery, Age variability, Sex variability, Strain variability	[10, 77, 79]
Subcutaneous, intraperitoneal, or intrahippocampal injection of KA	Rodent	Persistent SE	Repeated Intraperitoneal injections	[80, 82, 84]
Subcutaneous or intraperitoneal injection of pilocarpine	Rodent	Low cost	High mortality	[80, 82]
Subcutaneous or intraperitoneal injection of PTZ	Rodent	Easy procedure	Dose variability	[81, 83]

AEDs, antiepileptic drugs; KA, kainic acid; PTZ, pentylenetetrazol; SE, status epilepticus

## Temporal lobe epilepsy models

Temporal lobe epilepsy (TLE) is a result of an initial precipitating injury (IPI), such as SE, head trauma, or febrile seizures, with a latent period of weeks, years, or even decades after an IPI [89]. The process is divided into three main stages: (1) IPI, (2) latent period, and (3) TLE development [90]. TLE is the most common focal seizure with a complicated mechanism [84]. Hippocampal sclerosis is commonly observed in TLE, characterized by segmental neuronal cell loss in the CA1 and CA4 regions, astrogliosis, granule cell dispersion, and axonal reorganization [11, 71]. It is widely accepted that TLE mainly originates from the hippocampus and amygdala [91] and is occasionally accompanied by decreased cortical metabolism [92]. The optimal TLE model may meet several key points: (1) main origin from the hippocampus, amygdala, or other limbic brain regions; (2) spontaneous recurrent seizures, (3) similar pathological features mimicking human TLE, such as hippocampal sclerosis; (4) failure to control seizure attacks with classical AEDs; and (5) survival of model animals under effective plasma drug concentrations [93]. Electrical stimulus in the amygdala is a classical TLE animal model, which has been established in rats [94] and rhesus monkeys [95]. Electrical stimulus TLE models are also widely used to test targeted AEDs, with reliable seizure onset. However, this model is labor intensive, which is disadvantageous because of the complicated procedure and stereotaxic surgery [94, 95].

KA can combine with receptors on the postsynaptic membrane and induce excitatory postsynaptic electrical potential, inducing seizure attacks [96]. The seizure lesions induced by KA are mainly located on the edge of the hippocampus [97], perfectly mimicking the classical histopathological features of hippocampal sclerosis. Accordingly, the KA animal model is the most frequently used [96] and a potential refractory epilepsy animal model [98]. KA kindling can be classified as either local or systemic. Local administration refers to the intra-hippocampal, intraamygdala, or intracerebroentricular injection via a stereotaxic device, whereas systemic administration refers to subcutaneous or intraperitoneal injections [99]. Currently, TLE animal models have been established by intraperitoneal [100, 101], intracerebroventricular [96], intraamygdala [96], and intrahippocampal [96] injections. Systemic administration of KA leads to pathological changes, including loss of pyramidal cells in the hippocampal CA1, CA3, and CA4 regions, mossy fiber sprouting, neuron loss in the hilus, dispersion of the cell layer in the dentate gyrus, neurocyte atrophy in the midbrain, thalamus, substantia nigra, olfactory bulb, and entorhinal and piriform cortices [86]. The merits and demerits of systemic and local administrations are reviewed. Systemic administration is a simple procedure without craniotomy and cerebral trauma. However, its demerits are as follows: (1) neuronal damage not limited to limbic structures with more extrahippocampal damage, which contradicts the pathological features of TLE; (2) low KA bioavailability in the brain; and (3) higher mortality than local administration [84, 96, 102]. The merits of local administration are as follows: (1) KA directly entering the brain, giving rise to a higher bioavailability; and (2) induction of hippocampal sclerosis and spontaneous seizure attack, which better mimics the pathophysiology of TLE. Conversely, the demerits of this administration are as follows: (1) complicated procedure and (2) more likely induction of SE, even threatening life [102]. Altogether, previous KA models have several disadvantages: induction of SE, high mortality, and inconsistent latent period of spontaneous seizure attack. Recently, regarding the several points mentioned above, an improved model has been established via simultaneous injection of lorazepam and KA, with the following merits: (1) simple procedure, (2) regular latent period of 10-15 days, (3) avoidance of SE, and (4) lower mortality[102]. Similarly, a modified KA model was established by 2-5 graded intraperitoneal injections of KA (5 mg/kg) to male F344 rats (6-8 wks old) to induce SE, with diazepam to terminate convulsive seizures after 2 h of onset of SE. This model shows constant frequency and intensity of spontaneous recurrent seizures over days, weeks, and months, and impaired cognitive, memory function, and depressive-like behavior, with typical neuropathological features of chronic TLE. More importantly, it is ideal for testing AEDs or biologics with the potential to ease spontaneous recurrent seizures and comorbidities [103].

Pilocarpine, a cholinergic muscarinic agonist, activates acetylcholinergic receptors, leading to seizure attacks [104]. In 1983, pilocarpine was first identified to damage extensive brain tissues, such as the amygdala and hippocampus, via intraperitoneal injection, inducing seizure attack [86]. Recently, intraperitoneal injection of pilocarpine has become a popular TLE animal model [105]. Systemic injection of pilocarpine can cause cell injury in the hilus and dentate granule, neuronal degeneration in hippocampal CA1 and CA3 regions, and nuclei injury in the thalamus and amygdala[86]. In pilocarpine models, seizures usually originate from the hippocampus and spread to the amygdala and neocortex. Accordingly, the EEG results primarily show theta rhythm and isolated interictal spikes of the hippocampal region and the subsequently low voltage fast activity of the neocortex [86]. Additionally, animals with intrahippocampal or intracerebroventricular injection of pilocarpine can also present similar EEG results, behavior, and pathologic features to TLE [84]. Compared with intrahippocampal or intracerebroventricular injection, intraperitoneal injection presents a simple procedure without craniotomy, but varies in age, sex, and strain [86, 105]. In addition, extrahippocampal lesions are commonly observed. In contrast, intrahippocampal or intracerebroventricular injections are technologically demanding and time-consuming with lower mortality [84].

Similarly, coriaria lactone is a GABA antagonist that serves as a chemical epileptogenic agent [106]. Its repeated intramuscular injection can induce partial seizures in SD rats [107] and rhesus monkeys [106], eventually progressing to generalized tonic-clonic seizures and TLE [106, 107]. After coriaria lactone injection, a series of behavioral changes were observed, including the following stages: (1) hypomotoric; (2) staring, polypnea, and auricle vasodilation, (3) uncoordinated behavior, vibrissae twitch, facial clonus, general clonus, forelimb clonus, (4) bilateral forelimb clonus, and (5) poor balance, generalized tonic-clonic seizures, and generalized tonic phase [107]. The corresponding EEG changes are as follows: brief spikes, followed by polyspikes and focal spikes, epileptic discharges with high-amplitude, and episodic clusters of epileptiform discharges [107]. The coriaria lactone model can induce reliable seizure onset with a simple procedure and low mortality without craniotomy. Commonly, pathological changes consist of mitochondrial damage of neurons and glial cells, as well as degeneration and necrosis of hippocampal astrocytes. However the model is immature, occasionally no obvious structural changes have been observed, needing further verification [106, 107].

Theiler's virus infection chronically alters susceptibility to seizures [108]. After Theiler's virus infection, the mice experience acute encephalitic seizures with generalized behavioral seizures, characterized by sudden cessation of the ongoing behavior or sudden waking from sleeping, followed by limb clonus, recurrent rearing and falling, lethargy, extreme grooming, and wild running. The corresponding EEG recordings are pre-ictal spiking, spiking discharges with high frequency, post-ictal suppression, and post-ictal spiking with extended periods [109]. After the acute phase, the mice gradually progressed to chronic epilepsy. Altogether, the mice experienced acute symptomatic seizures, a latent period, and chronic spontaneous seizures in the course of the disease, mimicking the classical progress of TLE [109]. Additionally, intracerebroventricular injection of Theiler's virus can cause pathological changes similar to TLE, such as profound loss of pyramidal neurons in the hippocampal CA1 and CA2 regions, microglial activation, astrogliosis, apoptosis, and hippocampal inflammation [110]. Theiler's virus model is a window for viral-induced epileptogenic mechanisms. Compared with other virus models, such as the West Nile virus, measles virus, and HSV-1, Theiler's virus model presents a lower mortality rate. The main disadvantages are the unprecise latent period, poor seizure persistence due to the rapid clearance of the virus, and intracerebral inoculation location variability [109-111].

GABA can reduce neuronal excitability and synchronize the firing of projection neuron ensembles, known as the chief inhibitory neurotransmitter [112]. Tetanus toxin blocks GABAB receptors and inhibits dentate granule cells, inducing seizure attacks. Intrahippocampal injection of tetanus toxin is a newly established TLE model [97, 113-115]. After the unilateral intrahippocampal injection of tetanus toxin, bilateral and fast ripples with epileptic high-frequency activity can be recorded, with earlier occurrence in the ipsilateral hippocampus and later occurrence in the contralateral hippocampus. Fast ripples are also predominant in the dentate gyrus and the CA3 and CA1 regions [115]. Pathological changes vary according to the dose of tetanus toxin. A lower dose has lower mortality, although no obvious neuronal loss can be observed. Occasionally, atrophy and gliosis of the tissue as well as focal pyramidal neuronal loss in the hippocampal CA1 region may be found. In contrast, high mortality and substantial neuronal loss may be found at higher doses [84]. The tetanus toxin model is a window for GABA-related epileptogenic mechanisms with bilateral hippocampal epileptic activity. The disadvantages of this model include the absence of typical hippocampal sclerosis and neuronal loss, limited spontaneous seizures, and variability in inoculation location and dose [97, 113-115].

As a classical epileptogenic agent, penicillin is widely used in the establishment of animal models of epilepsy [116]. Sumbul and Aygun et al. have reported that the microinjection of penicillin into the sensorimotor cortex induced epileptiform activity in rats [117]. Additionally, the injection of penicillin into the hippocampus has established TLE models in macaques [118]. Penicillin injection can cause staring without movement, head leaning, oral automatism, chewing, lip smacking, and some manifestations similar to those presented by patients with TLE [118]. Accordingly, the related pathological changes include neuronal loss, glial cell proliferation, hippocampal sclerosis, decreasing synapses in the CA1 region, and many glial fibrillary filaments [118]. The penicillin model is reproducible with reliable seizure onset, typical electroclinical features, and no extrahippocampal lesions. Repeated injections are required for seizure attacks. Additionally, the simple mechanism involved in the model cannot mimic the complicated mechanism of humans [117, 118].

Febrile status epilepticus (FSE) can change the brain structure and function [119], increasing the probability to develop TLE [120]. Febrile seizures upregulate the GABAA receptors and induce a reversal in the direction of granule cell migration, increasing the susceptibility to limbic seizures and development of epilepsy [121]. Additionally, increased brain temperature leads to the activation of inflammatory pathways, and cytokine release may be responsible for the seizure attack [122]. Febrile seizure models usually present facial automatism, body tonic flexion, and respiratory alkalosis. The EEG results are also characterized by spike-waves and trains of spikes, mainly originating from the amygdala, hippocampus, and temporal cortex [46]. FSE can cause acute hippocampal injury, progressing to hippocampal sclerosis after 1 year [123]. A previous study induced generalized convulsive seizures in rats by exposure to 45-50 °C dry air, establishing the FSE TLE model [124]. Rodent FSE models are conducive to understanding the potential mechanisms of TLE [125]. The FSE model can induce seizure onset rapidly, with typical hippocampal sclerosis, but without spontaneous seizures [97, 119, 120, 124, 125].

Controlled cortical impact (CCI) models refer to the electromagnetic or pneumatic impacts that is compressing the exposed brain, causing brain injury with varying severity. The CCI models have been successfully established in ferret, mouse, rat, swine, and monkey, mimicking brain injury caused by cerebral trauma, with pathological changes in the gray matter, white matter, hippocampus, and thalamus [35, 36, 126, 127]. Except for seizure attacks, the CCI rat models mainly manifest as cognitive impairments and movement disorders. Movement disorders usually disappear within several months after CCI induction, whereas cognitive impairments may persist for a long time and are related to brain atrophy and progressively decreased cerebral blood flow. Moreover, emotional deficits have occasionally been reported needing further verification [36, 38]. Histopathological severity is positively related to the degree of impact velocity and cortical deformation [36]. Classical characteristics consist of cavitary lesions, hippocampal loss, and cortical surface injury ipsilateral to the impact region [35]. Additionally, some chronic pathological results, known as the classical characteristics of TLE (i.e., mossy fiber sprouting and delayed hippocampal lesions) have been observed. Accordingly, CCI models are qualified as potential TLE models [38]. The CCI model is reproducible, with a short potent period and spontaneous seizures. However, no study has proven that the CCI model is suitable for therapeutic predictive validity. Additionally, craniotomy, complex device, and mechanical variation must be considered [35, 36, 38, 126, 127].

Previous studies have established a hypoxic state by exposing postnatal day 11-12 male rat pups to intermittent asphyxia, successfully inducing electrographic seizures with convulsions [128]. Rodent hypoxia models can present spontaneous seizures and behavioral changes (e.g., aggressiveness, memory impairments, and social deficits). After hypoxia occurrence, EEG recording shows the reduced background amplitude first, followed by intermittent bursts of spike-wave and suppression, followed by spike-wave with regular rhythm, which subsequently becomes frequent and complicated with the occurrence of polyspike waves [129]. Regarding pathological changes, mossy fiber sprouting, abnormal excitability, and plasticity of hippocampal networks have been observed [46]. Mossy fiber sprouting is a hallmark of TLE; therefore, the hypoxia model has the potential to be a candidate for TLE models [130]. Hypoxic ischemic encephalopathy is one of the most frequent reasons of neonatal seizures[131]. Type 2 K^+^-Cl^-^ cotransporter is closely related to potential pathophysiology of hypoxic ischemic encephalopathy [132]. Hypoxia-ischemia exposure induced both generalized and focal seizures in neonatal mice with a high induction rate and variability in lesion size and behavioral deficits [129]. Hypoxia-ischemia models are also cost-efficient and easy to reproduce with simple procedures and high induction rates. However, high mortality, limited induction of spontaneous seizures, and age, strain, and behavioral deficit variability should be considered when performing the induction of seizures [129, 130, 133]. The associated advantages and disadvantages of all the TLE models mentioned above are listed in Table 6.

**Table 6:** Temporal lobe epilepsy models.

Model	Animal	Advantages	Disadvantages	References
Electrical stimulation of the amygdala	Wistar male rats; Monkeys	Reliable onset, Test AEDs	Stereotaxic surgery, Complicated procedure, Labor-intensive	[94, 95]
Subcutaneous or intraperitoneal injection of KA	Rat	Simple procedure, No craniotomy	Extrahippocampal injury, Low bioavailability, High mortality	[84, 96, 102]
Intracerebro-ventricular, intraamygdala, or intrahippocampal injection of KA	Rat	High bioavailability, Hippocampal sclerosis, Spontaneous seizure	Complicated procedure, Convulsive SE, High mortality	[84, 96]
Subcutaneous injection of KA and lorazepam	Sprague-Dawley rats	Simple process, Predictable latent period, No convulsive SE, Low mortality	No convulsive seizures, No comorbidities, Novel model	[102]
Graded intraperitoneal injections of KA	F344 rat	Spontaneous recurrent seizures, Cognitive and mood impairments, Typical neuropathology Test AEDs	No evaluation for female rats, No evaluation for older rats	[103]
Intraperitoneal injection of pilocarpine	Rat Guinea Pig Mice	Simple procedure, No craniotomy	Age variability, Sex variability, Strain variability, Extrahippocampal lesions	[86, 105]
Intrahippocampal or intracerebro-ventricular injection of pilocarpine	Mice	Low mortality, Typical behavioral changes, Typical EEG and histopathology	Technologically-demanding, Time-consuming	[84]
Intramuscular injection of coriaria lactone	Rhesus Monkey SD rat	Simple procedure, No craniotomy, Reliable onset Low mortality	Need further verification	[106, 107]
Intracerebro-ventricular injection of Theiler's virus	C57BL/6 mice	Lower mortality, Viral-induced, Epileptogenic mechanism	Stereotaxic surgery, Inoculation location variability, Unprecise latent period, No persistent seizure	[109-111]
Intrahippocampal injection of tetanus toxin	Mouse Rat	Bilateral hippocampus epileptic activity, GABA-related mechanism	Limited spontaneous seizures, Dose variability, Inoculation location variability, No hippocampal sclerosis, No neuronal loss	[97, 113-115]
Intrahippocampal injection of penicillin	Macaques Rat	Reproducible Reliable onset, No extrahippocampal lesion, Typical electro clinical features	Simple mechanism, Repeated injections	[117, 118]
Febrile	Sprague-Dawley rat C57BL/6J male mice	Rapid onset, Hippocampal sclerosis	Limited spontaneous seizures	[97, 119, 120, 124, 125]
Controlled cortical impact	Ferret, mouse, rat, swine, monkey	Short potent period, Spontaneous seizures, Reproducible	Craniotomy Complex device, Mechanical variation, No therapeutic predictive validity	[35, 36, 38, 126, 127]
Hypoxia/ischemia	Rat	Simple procedures, Low cost, Reproducible, Reliable onset	Strain variability, Age variability, Lesion size variability, High mortality, Fewer spontaneous seizures	[97, 129, 130, 133]

AEDs, antiepileptic drugs; EEG, electroencephalogram; GABA, γ-aminobutyric acid; KA, kainic acid; SE, status epilepticus

## Summary

Currently, most animal models have been established for pediatric or young patients, but not for older patients [134]. The literature shows that a higher age gives rise to a higher hippocampal excitability and a higher risk of developing spontaneous seizures [135]. The elderly often have comorbid conditions, conveying a greater predisposition to the first seizure [136]. Additionally, they have to take more medications because of the comorbid conditions, leading to complicated multi-drug interactions and altered responsiveness to AEDs [134]. Inconsistent pathological changes in young and aged rat models have also been observed [137]. Aged animal models should receive more attention [138]. Moreover, compared with acute seizure models, chronic seizure models are more suitable for mimicking neuropathology and behavioral comorbidities that patients often experience. Accordingly, chronic seizure models are more appropriate for identifying promising anticonvulsive compounds [138]. Furthermore, seizure may manifest as a secondary symptom of cerebrovascular diseases, such as cerebral venous thrombosis [139] and neonatal stroke [140], or some neurodevelopmental diseases, such as CDKL5 deficiency disorder [141]. In these diseases, seizure may cause severe consequences and even threaten life, despite the existence of other symptoms. Accordingly, secondary seizure models may be established from the perspective of seizure control in necessity.

Animal models are vital for research on the pathophysiology, potential mechanism, development and selection of AEDs, and assessment of the adverse effects of AEDs. Given the high heterogeneity of patients and the complexity of multiple seizure phenotypes and comorbidities, animal models cannot completely mimic real-world human seizure attacks, despite the continuous improvement of models. Altogether, combining concrete needs with comprehensive consideration of various factors of the models are vital for the selection of appropriate animal models. Meanwhile, continuous improvement of existing models and the development of new models are necessary for future research.
